# Tonic immobility and phenomenal consciousness in animals: a review

**DOI:** 10.3389/fpsyg.2025.1509999

**Published:** 2025-02-28

**Authors:** Michael L. Woodruff

**Affiliations:** Department of Biomedical Sciences, Quillen College of Medicine, East Tennessee State University, Johnson City, TN, United States

**Keywords:** tonic immobility, death feigning, anthropomorphism, intentional states, fear, phenomenal consciousness, predator imminence theory, theory of mind

## Abstract

Tonic immobility (TI) is an innate, last-resort response to the presence of a predator, commonly referred to as feigning death or thanatosis. However, it is critical to distinguish death feigning from TI; the former encompasses a complex series of behaviors, with TI representing its final aspect. Given this complexity, death feigning is hypothesized to manifest a higher-order intentionality in animals. Considering that third-order and higher intentional states are correlated with some form of phenomenal consciousness, their presence in animals has significant implications for their conscious experiences. This hypothesis surrounding third-order and higher intentional states is subject to dispute, in part due to the lack of sufficient evidence indicating that the behaviors associated with death feigning, aside from TI, serve a protective function against predation. Evidence suggests that TI alone constitutes an effective mechanism for predator defense. It is posited that the cessation of TI by the prey organism signifies the presence of a first-order intentional state. Furthermore, it is proposed that behavioral indicators suggest that the termination of TI by the prey is linked to anoetic and potentially noetic consciousness. The relationship between TI and fear is also examined. It is contended that, within the framework of formulating hypotheses concerning the mechanisms of TI and developing experimental designs to test these hypotheses, fear should be characterized as an intervening variable. The conclusions derived from this analysis indicate that TI can occasionally function as an effective predator defense associated with primal sensory and anoetic consciousness. Its termination may imply the presence of noetic consciousness; however, self-reflective autonoetic consciousness appears to be absent. Finally, the hypothesis suggesting that TI serves as an evolutionary precursor to the theory of mind in humans is discussed, serving as a cautionary note in the interpretation of findings from animal research concerning the evolution of cognitive functions in humans.

## 1 Introduction

Tonic immobility (TI) is an innate defensive response against predators (Fanselow and Lester, 1987; Gallup, 1977; Ratner, 1967; Woodruff, 1977). In species that exhibit TI, the primary triggering stimuli are physical restraint and pressure on certain body parts. The overt responses to these stimuli include a loss of the righting reflex and immobility that persists beyond the restraint (Carli, 1977; Carli and Farabollini, 2022a; Gallup, 1974a; Klemm, 1977). The simplest explanation for TI is that it represents a reflexive response to intense tactile and proprioceptive stimulation (Klemm, 1971). If TI serves as a defense against predators, it does so because immobility reduces the stimuli that provoke predators to attack prey (Gallup and Rager, 1996; Herzog and Burghardt, 1974; Kaufman, 1974; Sargeant and Eberhardt, 1975; Thompson et al., 1981; Van Hemel and Colucci, 1973). This interpretation does not require the assumption that the prey intentionally feigns death, that the predator perceives the motionless animal as deceased, or that the prey experiences fear.

Alternative, more cognitively oriented hypotheses have been proposed. For example, Burghardt (1991) identifies TI as the final behavior in death feigning by the Eastern hognose snake. Given that death feigning is a flexible and complex behavioral sequence, Burghardt dismisses a simple behavioristic explanation for it. Rather, following Donald Griffin’s theoretical position (Griffin, 1998; Griffin, 2001), death feigning is, as the name suggests, a purposeful deceptive act. In other words, the snake engages in death feigning, ultimately entering TI, because it wants the predator to believe it is dead (Burghardt, 1991, p. 76). Therefore, for Burghardt, death feigning indicates the presence of an intentional state—specifically, a mental state that refers to, or is *about,* some object or state of affairs (Dennett, 1983; Jacob, 2023). Intentional mental states encompass beliefs, desires, and perceptions. Thus, according to Burghardt, the Eastern hognose snake does not merely perceive a predator; it also believes that the predator is dangerous and that purposeful death feigning reduces the threat.

Susana Monsó posits a connection between death-feigning behaviors and intentionality; however, she accentuates the intentional mental state of the predator (Monsó, 2021; Monsó and Osuna-Mascaró, 2021). She asserts that predatory animals cease their attacks on prey that have feigned death due to their belief that the animal has indeed succumbed to death. Furthermore, she contends that the act of death feigning suggests that at least a subset of animals possesses an understanding of the concept of death.

The death-feigning behavior, detailed below for the Eastern hognose snake, generally involves postural changes and various parasympathetic reactions. Immobility appears as the final stage of the display. Two questions arise regarding the association of death feigning with intentionality. One question is: what empirical evidence supports such an association? Another question is: if such an association exists, what type of intentionality is implied? It is one thing to assert that an unlearned, species-typical behavior indicates first-level intentionality, in which an animal perceives a predator as dangerous. It is another to claim that this behavior signifies a third-order intentional state, where the prey desires the predator to believe it is dead, and this belief prompts the predator to stop its attack (Burghardt, 1991; Monsó, 2021; Monsó and Osuna-Mascaró, 2021). I will explore these questions, starting with a discussion of death feigning and TI. Both are circa-strike predator-defense behaviors, but I propose they should be distinguished from the perspectives of behavioral topography and evolutionary history. Section 4 provides a review of experiments testing the hypothesis that either the death-feigning behavior or TI alone enhances a prey animal’s chances of escaping death by predation. This review concludes that TI provides some protection from predator-inflicted serious harm or death, while the other behaviors associated with the death-feigning behavior contribute little or nothing to this protection.

This conclusion is significant for evaluating the hypotheses proposed by Burghardt and Monsó. First, they assert that the presumed effectiveness of death feigning stems from the predator’s belief that the prey is dead. Second, the entire death-feigning behavior is necessary for the prey to convince the predator of this belief. TI alone is not sufficient. Therefore, the discussion will revisit these hypotheses in Section 5. It will be argued that the influence of environmental variables on TI offers empirical support for the assertion that 0-level and 1st-level intentional systems, as outlined by Dennett (1983), are linked to the initiation and termination of TI, respectively.

Additionally, the association of TI with an intentional state suggests a reason to explore the potential relationship between TI and consciousness. Although, as Gennaro (2018) notes, there is disagreement among philosophers regarding the precise relationship between intentionality and consciousness, it appears likely that, while not all intentional states are conscious (Dennett, 1983; Searle, 1979), intentionality and phenomenal consciousness are connected (Siewert, 2022) to the extent that phenomenal consciousness requires intentionality, if not vice versa (Gennaro, 2018, p. 108). Therefore, a discussion of the possible relationship between TI and phenomenal consciousness is presented in Section 5.

In this discussion, phenomenal consciousness will be defined as the experience of the quality of exteroceptive and interoceptive sensations, emotions, motivations, and memories, along with the possible sense that there is something that it “feels like” to have them (Allen and Trestman, 2024). A concern in this discussion will be the question: if a relationship between TI and phenomenal consciousness exists, what type of phenomenal consciousness is indicated? It is one thing to propose that TI indicates unreflective primal sensory consciousness and another to claim that it signals some higher-order consciousness requiring self-reflective awareness.

A second reason to explore the potential relationship between TI and phenomenal consciousness arises from the prevailing hypothesis that fear is invariably linked to TI (Carli and Farabollini, 2022b; Gallup, 1977; Jones, 1996). If TI functions as a measure of a subject’s fearfulness, and fear itself is fundamentally a subjective experience (e.g., LeDoux, 2013; LeDoux, 2014; LeDoux and Hofmann, 2018), then TI may indicate phenomenal consciousness as defined earlier. This hypothesis will be analyzed in Section 6. This section will conclude with a discussion of Gallup’s (1977); Gallup (1979) suggestion that fear, when employed in formulating hypotheses about TI and designing experiments to test these hypotheses, is most effectively defined as an intervening variable in a manner that does not necessarily require a subjective state.

## 2 Distinguishing between TI and death feigning

TI and death feigning are sometimes defined similarly, as they both involve immobility and relative unresponsiveness to environmental stimuli (e.g., Carli and Farabollini, 2022a; Konishi et al., 2020; Rogers and Simpson, 2014; Sargeant and Eberhardt, 1975). However, if TI and death feigning are indeed the same behaviors, there appears to be no reason to retain the term “death feigning” along with its anthropomorphic implications. Nevertheless, many publications (e.g., Burghardt, 1991; Gabrielsen and Smith, 1985; Monsó and Osuna-Mascaró, 2021) clearly differentiate between them, describing death feigning as a complex display with TI as a subsequent component. Therefore, it may be that TI and the behavioral display known as death feigning (also referred to as thanatosis) can and should be distinguished.

As described by Carli and Farabollini (2022a, p. 331), TI is defined as “an all-or-none innate, reversible, reflex immobility response occurring in a prey/predator context, characterized by immobility and unresponsiveness.” Thus, TI represents a relatively straightforward motor response. Strong tactile and proprioceptive input, generated when a predator restrains its prey, is typically necessary to induce it (Francq, 1969; Gabrielsen and Smith, 1985; Klemm, 1966; Klemm, 1977; Norton et al., 1964; Sargeant and Eberhardt, 1975; Woodruff, 1977; Zidarova, 2022; Woodruff and Baisden, 1985).

Conversely, death feigning is a complex display that culminates in TI. A description of this display in the Eastern hognose snake provides an example: “If touched (and sometimes even when not touched), the snake may begin erratic writhing behavior—often frenetic and violent—and may even bite itself in the process. It defecates (or discharges its cloacal sacs), coils its tail like a pig’s, turns over, and finally becomes quiescent: mouth open, tongue extruded, blood coming from the mouth, and with no visible breathing” (Burghardt, 1991, p. 62). Thus, the Eastern hognose snake exhibits several behaviors associated with its death feign before entering TI. As discussed in subsequent sections, this is also true of death feigning in the Virginia opossum (Francq, 1969; Gabrielsen and Smith, 1985).

Additionally, death feigning not only represents a more complex display than TI but also exhibits a more limited species distribution. This practice is most commonly observed in snakes (Burghardt, 1991; Magallón et al., 2021; McDonald, 1974) and in some species of opossums (Francq, 1969; Gabrielsen and Smith, 1985; Kimble, 1997). Conversely, TI has a species distribution that spans the entire cladogram (Peinkhofer et al., 2021). This indicates that the evolutionary pressure to develop the behaviors immediately preceding TI in death feigning had a limited distribution. Therefore, as Burghardt (1991) suggests, the death feign may be a more recent evolutionary development compared to TI. Finally, as discussed in Section 4 and acknowledged by Burghardt (1991), there is no published evidence indicating that the pre-TI death feign is effective as a predator defense, while evidence shows that TI alone serves as an effective predator defense.

## 3 Predator imminence theory

Death feigning and TI are not isolated behaviors; they are part of a series of defensive reactions to predator behavior. Therefore, understanding them requires knowledge of the broader interaction between predator and prey. As summarized by Humphreys and Ruxton (2018), predators must first detect and identify their prey. If the prey is at a distance, the predator needs to close that gap. If the predator catches the prey, it must subdue it. Once subdued, the predator may or may not immediately consume the prey. Ratner (1967) proposed a series of distance-related defensive reactions that prey exhibit in response to these predator behaviors. A prey animal becomes immobile or “freezes” when it detects a potential threat from a distance. This helps reduce the stimuli used by the predator to detect and identify the prey (Gallup and Rager, 1996; Herzog and Burghardt, 1974; Kaufman, 1974; Van Hemel and Colucci, 1973) and may enhance crypsis in some species (Carli and Farabollini, 2022c; Caro, 2014). As the distance between the predator and prey decreases, the prey eventually flees. If caught, the prey struggles and fights back. Ratner (1967) did not mention the death-feigning behavior but included TI as a final defensive behavior if the previous actions were unsuccessful.

Fanselow and Lester (1987) integrated Ratner’s predator defense model into their predator imminence theory. According to Fanselow and Lester, the perception of imminence is influenced not only by the physical distance between predator and prey but also by the prey’s assessment of the time it would take for the predator to reach it and the proximity of refuge (Fanselow, 1994; Perusini and Fanselow, 2015). This theory outlines several modes of prey behavior, beginning with the pre-encounter mode, during which no specific predator is present, but the prey modifies its behavior based on previous experiences. For example, wild rabbits have been observed to prefer grazing in areas where they have encountered fewer predators historically compared to nearby areas with higher concentrations of predators (Monclús et al., 2009).

The post-encounter mode occurs when a prey animal detects a predator. Freezing in place and fleeing are the two most commonly described post-encounter behaviors. The choice between the two depends on the perceived risks associated with the situation, including whether the prey is aware of an escape route (Blanchard et al., 1976; Stankowich and Blumstein, 2005). Freezing is often accompanied by increased vigilance, which not only decreases the likelihood that the predator will notice the prey but also provides time to evaluate potential escape routes. Freezing is typically followed by flight as the predator approaches.

Circa-strike is the final mode in the predator imminence cascade, occurring just before, during, and immediately after physical contact with the predator. This mode is characterized by innate, species-specific defensive behaviors (Fanselow and Lester, 1987; Roelofs, 2017). Vocalizations and jump attacks exhibited by a wild rat when an experimenter approach (Blanchard et al., 1986) exemplify circa-strike behaviors.

The Eastern hognose snake and the Virginia opossum exhibit circa-strike behaviors that vary as a potential predator approaches their prey. For example, when a potential predator first enters its circa-strike zone, an Eastern hognose snake undergoes the “bluff” phase of predator defense (Burghardt, 1991). It raises its head and flattens its neck to appear larger, resembling a venomous viper while hissing loudly. As the predator continues to advance, the snake may strike (Burghardt, 1991; Greene, 1988; Young and Lalor, 1998). If the predator makes physical contact, the hognose often feigns death, twisting and turning its body while simultaneously defecating and discharging its cloacal sacs (Burghardt, 1991). Just before entering TI but while still writhing, the hognose frequently regurgitates and retches continuously; bradycardia occurs, and the hemipenis everts in male specimens (McDonald, 1974). These autonomic reactions persist even after the snake enters TI.

In the Virginia opossum, circa-strike responses initiate when a potential predator approaches within approximately two meters of the animal. These responses include baring the teeth, hissing, and growling—somatic reactions that are best classified as aggressive behavior. However, the release of flatulence, urination, defecation, and secretion from the paracloacal glands occurs simultaneously with these somatic responses. Similar to the Eastern hognose snake, these autonomic reactions indicate activation of the parasympathetic nervous system, likely associated with a reflex transition from active to passive predator defense behavior. When the opossum enters TI, the previously mentioned autonomic reactions continue, and heavy salivation begins. In males, the penis becomes erect (Francq, 1969; Gabrielsen and Smith, 1985), and the heart rate slows (Kimble, 1997).

The timing of the initiation of death feigning in response to predator behavior situates it within the circa-strike phase of the predator imminence cascade. However, the question of whether death feigning and/or TI provide protection from predation, as proposed for circa-strike behaviors, remains an independent issue. The outcomes of research investigating the efficacy of death feigning and TI as defenses against predators will be explored in the subsequent section.

## 4 Does feigning death or TI provide protection from predation?

### 4.1 Lack of evidence that death feigning protects against predation

Monsó argues that death feigning exists “because there is an advantage, not just to staying still, but specifically to appearing dead” (Monsó and Osuna-Mascaró, 2021, p. 2270). In her description of death feigning, Monsó highlights the autonomic responses and posture of opossums engaged in this behavior, illustrating the benefits of death feigning compared to TI alone with the following scenario.

A coyote attacks an opossum that exhibits death-feigning behavior. “The canine, though, would rather feast on fresh meat than on some long-dead corpse, rotting and full of pathogens. And so the danger passes. Then the opossum springs back into action, unscathed and unfazed, and goes about the rest of her day.” (Monsó, 2021, p. 1)

This is a nice story, but unfortunately, Monsó offers no empirical scientific studies that demonstrate death feigning provides any protection from predation. Instead, she appears to depend on the presumed general validity of the adaptationist (selectionist) paradigm. This paradigm is, at least implicitly, accepted by many biologists and most evolutionary psychologists. According to it, every trait evolved because its function provided a selective advantage to the organisms exhibiting it. However, in the absence of empirical evidence that adaptation through natural selection accounts for a specific behavior, alternative hypotheses, such as the constructive neutral evolution (CNE) theory, should be explored. CNE suggests that complex systems can evolve through neutral genetic drift without conferring a fitness advantage over their simpler ancestors (Muñoz-Gómez et al., 2021). Therefore, rather than assuming that death feigning has an adaptive function, it appears appropriate to quote evolutionary biologist Koonin (2016, p. 6), who stated, “Arguably the most realistic approach is to assume a neutral null model and then seek evidence of selection that could falsify it.” In other words, one should hypothesize that death feigning and TI are not adaptive and then design experiments to test this hypothesis.

Evidence suggesting that the neutral null model may be rejected for TI is reviewed in sections 4.2, 4.3, 4.4, and 7.4. However, experiments involving the Virginia opossum, discussed in section 4.3 below, support the neutral null model for most aspects of the death-feigning behavior. Aside from terminal immobility, the responses associated with death feigning did not deter dog attacks on the opossums. In fact, the effectiveness of death feigning as a defense against predators has long been questioned by biologists (Edgren, 1955; Edgren and Edgren, 1955). Honma (2021, pp. 25-26) expresses these doubts as follows:

“It is unreasonable to assume that the function of TI is to escape attack from a predator by becoming immobile as if it were dead if one takes the point of view of the predator. TI is triggered when a predator approaches or makes contact with a prey to capture it. In that case, the predator is considered to have already detected and recognized the prey as food. Therefore, for the predator avoidance behavior as described above to work, we must assume a situation in which the predator abandons the food it caught because it has just died.”

Furthermore, in what appears to be the only published account of a predator reacting to death feigning, McDonald reports the results of a staged encounter between a specimen of *H. platyrhinos* and a *Lampropeltis getulus holbrooki* by placing them in a large aquarium. As soon as the kingsnake approached, the Heterodon displayed typical behavior, first attempting to intimidate the kingsnake and then writhing and feigning death. Without hesitation, the kingsnake attacked and devoured the unresisting Heterodon (McDonald, 1974, p. 162).

An isolated observation does not, by any means, disprove a hypothesis. However, McDonald’s report should prompt caution regarding the acceptance of speculative narratives about the effectiveness of the complex death-feigning behavior as a mechanism for predator defense. Nevertheless, despite the lack of evidence suggesting that the pre-TI death feign provides protection from predation, findings from both naturalistic and semi-naturalistic laboratory studies support the hypothesis that merely engaging in TI without accompanying death feign behavior may reduce the likelihood of becoming preyed upon by predators.

### 4.2 TI as a defense against predation on ducks by foxes

Naturalistic studies of TI in insects provide substantial direct evidence for the hypothesis that TI provides protection against predation (Carli and Farabollini, 2022c; Sakai, 2021). However, the generality of this hypothesis will be enhanced if research demonstrates TI’s effectiveness as a defense against predation in vertebrate species. Such research is understandably challenging to conduct. Unstaged field observations of vertebrate predator–prey interactions are rare, and when they do occur, the presence of a human observer may disrupt the normal pattern of interaction. Moreover, contrived predator attacks on prey raise significant ethical concerns. With this caveat in mind, I will describe such studies in the following subsections.

The most convincing study by Sargeant and Eberhardt (1975) observed the interaction between red foxes and ducks. Initially, the ducks fled from the foxes, and while some escaped, 50 were caught in a fox’s jaw. These ducks became immobile, and 29 survived because the fox either laid the motionless duck down and wandered away, cached it under loose vegetation, or left it to pursue nearby moving ducks. The following excerpt from Sargeant and Eberhardt illustrates an example.

An adult male fox attacked two female mallards standing on a graveled roadway in the pen. The fox seized one, and the bird death-feigned immediately. The fox then walked about 15 m and laid the bird down. After mouthing it, he started back toward the second bird but turned and saw the first bird escaping. He ran back, recaptured it, and the bird death-feigned again. The fox laid it down again at the same site, mouthed it, and began walking back toward the second bird. Midway between the two birds, he turned and saw the first bird escaping again. The fox hesitated, but this time, attacked and captured the second bird. The first bird escaped to the pond. (Sargeant and Eberhardt, 1975, pp. 113-114)

When approached by a fox, the ducks did not perform an elaborate display similar to the death feigning observed in opossums and hognose snakes; instead, they simply became immobile. Thus, while Sergeant and Eberhardt assert that the ducks are “death-feigned,” their reaction to being seized is more accurately described as TI, supporting the hypothesis that TI alone functions as a predator defense. It also demonstrates that foxes prefer to attack moving ducks rather than those that are immobile and that the fox’s behavior is influenced by varying stimulus conditions. This understanding of effective predator defense does not require that the prey engage in a deliberately deceptive display or that the predator believes the prey is dead.

### 4.3 TI and attacks on opossums and a polecat by dogs and humans

Trauma-induced immobilization (TI) resulting from dog and human attacks has been observed in the Virginia opossum. Norton et al. (1964) used either a steel “dog jaw” resembling a large pair of pliers wielded by humans or real dogs to study TI in these animals. The experimenter grasped the opossum with the artificial jaws and shook it vigorously. Before entering TI, the opossum “would typically enter a rage state, hissing, growling, and attempting to attack the artificial jaw.” (Norton et al., 1964, p. 162). In a second experiment, Norton et al. allowed a dog to chase an opossum into a corner, where it picked it up by the neck and shook it. The opossum quickly entered TI, and the dog dropped it.

Francq (1969) also observed the behavior of opossums when attacked by either a human or a dog. The human initially “harassed” the opossum by yelling, clapping, and poking the animal’s head with a stick. He then seized it by the nape and sacral area and shook it. The dog barked while approaching the opossum, then nipped at it before attempting to secure a bite on the nape. The opossums entered TI in 67% of the trials when the humans captured them. In contrast, the opossums entered TI 100% of the time when the dog seized them. This observation suggests that the opossum perceived the dog as more threatening than the human and that the dog’s teeth were more effective in providing the necessary tactile stimulation to induce TI than human hands. Once the opossum entered TI, the dog would drop it on its side.

According to Francq, opossums adopted a semirigid posture during TI. Their tails curved ventrally, their eyes and mouths remained open, and their tongues extended through their teeth. The animal salivated, urinated, and, similar to their defensive display prior to being seized, males unsheathed their males. Opossums of both sexes defecated and released a strong-smelling green mucus from their paracloacal glands during TI.

Two experiments conducted by Gabrielsen and Smith (1985) produced results consistent with those found by Francq (1969). In the first experiment, the behavior of the opossum was evaluated when a dog was at a distance. The investigator walked the dog at heel toward the opossum, stopped 2 m away, and then turned to walk back. The opossums became motionless in response to this perceived threat. In the second experiment, the dog was led to the opossum and allowed to grab it by the nape and shake it. The opossums quickly entered TI once the bite on the neck occurred. The authors’ description of the opossum in TI matches that of Francq (1969).

According to predator imminence theory, the behavior of the opossums reported in these publications during the entire attack depended on the actions of the predator, whether it was a human or a dog. When the predator approached within about 5 m of the opossum, it would flee. If the predator got closer than approximately 2 m, the opossum would turn, growling, and face the predator with its mouth open and teeth bared. When the predator circled, the opossum would rotate to maintain its gaze on it. During this phase of the threat response, the opossum released flatulence, defecated, and emitted a strong-smelling, greenish, mucus-like substance from its paired paracloacal glands. Thus, the opossums exhibited several responses that are also described as part of death feigning. These responses, therefore, are not unique to feigned death.

Unfortunately, these studies provide only vague descriptions of the dogs’ reactions to the opossums. However, the dogs did drop or lay the immobile opossums down when they entered TI. Furthermore, Francq (1969) observed that one dog showed no interest when prompted to resume its attack on an immobile opossum. A similar observation was made by Gabrielsen and Smith (1985, p. 395), who reported: “Despite our strongest encouragement, our dog invariably lost interest in the opossum once it entered this state.”

Finally, to broaden the observation of TI to a species typically classified as a predator, TI was incidentally observed in response to being seized by a dog in a marbled polecat (*Vormela peregusna*) during a field survey of the distribution of this carnivorous weasel in western Bulgaria (Zidarova, 2022). The dog dropped the polecat upon command, making it impossible to determine whether TI would have caused the spontaneous release of the polecat without inflicting lethal damage. However, after the dog and the surveyor moved away, the polecat righted itself and quickly ran for cover, indicating that it remained vigilant during the TI episode.

### 4.4 TI and attacks on quail by cats

Thompson et al. (1981) tested the hypothesis that cats would preferentially attack moving Japanese quail rather than quail in TI. A quail induced into TI by an experimenter was placed at one end of an arena, while a mobile quail was positioned at the opposite end. A cat was released at the far end of the arena. In 14 out of 16 trials, cats chose the freely moving bird, thereby supporting the hypothesis. This outcome also suggests that the survival value of TI may be enhanced when mobile prey animals are nearby.

In a second experiment, two fully mobile quails were placed at the end of the arena before a cat was released. The total time the cat spent stalking, attacking, and handling each bird was recorded, as well as the time the quail spent either moving or remaining frozen when the cat was at a distance or in a state of immobility after being captured. Four cats served as predators. Two of the cats only stalked the birds, which did not enter a state of immobility; they fled from the cats, except for one quail that froze in place for 100 s, during which the cat ignored it and stalked the other moving bird. The other two cats attacked and captured quail with their mouths. Analysis of the data indicated that the more time a quail spent in a state of immobility, the less time it was preyed upon. These results suggest that immobility, in the absence of any other response, reduces the active molestation of prey by predators.

In summary, the reports by Gabrielsen and Smith (1985), Francq (1969), Norton et al. (1964), Sargeant and Eberhardt (1975), and Zidarova (2022) strongly indicate that several species of vertebrates exhibit TI when captured by a predator. Furthermore, the observations made by Sargeant and Eberhardt (1975) provide direct support for the hypothesis that TI serves as a viable predator defense on its own. Additionally, the results published by Thompson et al. (1981) and Sargeant and Eberhardt (1975) suggest that predators are less likely to attack immobile prey compared to moving prey. This holds true whether the predator has observed the prey from a distance or has already captured it and simply laid it down. These studies also demonstrate that a more complex death feign is unnecessary to reduce the likelihood of molestation by a predator.

## 5 Intentionality, death feigning, and TI

### 5.1 The intentional stance and death feigning

Publications discussed in the previous section suggest that, aside from the terminal TI response, the behaviors associated with death feigning do not provide protection from predation. However, given the lack of published research on this topic, a saying likely originating as far back as the 1880s should be noted: “The absence of evidence is not evidence of absence.” It is reasonable to assume that replications of McDonald’s (1974) experiment would demonstrate that death feigning does offer some protection from predation for the hognose snake, particularly against carnivorous mammals like raccoons and opossums, even if it does not protect against larger snakes. This possibility gives us one reason to discuss Burghardt’s (1991) proposal that death feigning reflects the presence of intentional states (Dennett, 1983) in animals. A second reason is that, while pre-TI death-feigning behaviors may not exhibit intentionality in animals, the shift from TI to escape behavior might.

Burghardt (1991) uses the term “intentional” as defined by philosophers. Intentionality refers to the quality of a mental state that references or concerns *an* object or a state of affairs (Dennett, 1983; Jacob, 2023). Intentional mental states encompass beliefs, desires, and perceptions. From this viewpoint, the Eastern hognose snake perceives a predator and possesses a mental representation that includes the belief that it is dangerous. This belief drives the decision to intentionally engage in death-feigning behaviors, as the snake considers these actions will decrease the threat. Or, as Ristau (1991) might articulate, the snake makes a deliberate choice to feign death to attain the desired objective of safety.

To substantiate the hypothesis that death feigning indicates an intentional mental state, Burghardt (1991) reviewed various experiments in which environmental conditions deemed to signify threat or safety were systematically manipulated. However, there is a significant issue with the examples he presents. With the exception of a study by Burghardt and Greene (1988), none of the referenced experiments include responses related to death feigning, aside from TI, as dependent variables. Instead, the dependent variables primarily consist of the frequency and duration of successful inductions of TI. Indeed, in the study by Burghardt and Greene (1988), the only noteworthy effects relate to the duration and cessation of TI, with no other behaviors associated with death feigning demonstrating any significant influence. Consequently, these experiments fail to substantiate Burghardt’s hypotheses; however, they may support the hypothesis that an intentional mental state within prey affects the cessation of TI.

### 5.2 Intentionality and TI

This hypothesis is best analyzed within the framework of predator imminence theory. The transition from one mode of predator defense behavior to another indicates that the prey makes a decision that impacts its well-being. For example, the shift from the pre-encounter mode, during which the animal engages in foraging, to the post-encounter mode, wherein it may either freeze or flee, signifies a preference for stimuli correlated with safety rather than nutrition. In the post-encounter mode, the choice to freeze or flee is influenced by variables such as the distance between the predator and the prey, as well as the prey’s awareness of potential escape routes (Blanchard et al., 1976; Stankowich and Blumstein, 2005).

These choices require a risk assessment conducted by the prey animal. According to Blanchard (2018, p. 70), risk assessment is defined as a process that “facilitates the acquisition of information about threats and the situations in which they occur, allowing (if successful) the determination of an optimal defense for that particular threat stimulus and situation, including a return to non-defensive behavior if the threat is absent.” Throughout the risk assessment process, it’s reasonable to infer that the prey has mental representations of both the predator and the environment. Based on these mental representations, the prey forms beliefs about the predator (e.g., its danger level, its speed) and the environment (e.g., the presence of nearby bushes as a potential refuge). These beliefs significantly influence the decision-making process. Consequently, the hypothesis that the prey animal has intentional states clarifies the methodology it employs in making choices. Moreover, the outcomes of systematic manipulation of environmental stimuli in laboratory experiments may extend this argument to include the concept of Tactical Integration (TI).

Prior to the presentation of these experiments, it may be helpful to describe the general procedure for studying TI in a laboratory setting. Except for a study conducted by Burghardt and Greene (1988), the experiments detailed below made slight modifications to the following methodology. Following removal from its home cage and subsequent transport to the testing site, the subject is handled by the experimenter—using either one or two hands, depending on the species—often in an inverted orientation and placed onto a stable surface. The subject is then manually restrained for a duration typically ranging from 15 s to one minute, after which the restraining hand is gently released. In most instances, the subject must remain immobile for a period of 10 to 15 s following the removal of restraint for the induction attempt to be classified as successful. Generally, between 3 and 10 attempts are made to induce TI within a single experimental session, with the number of successful inductions serving as one dependent variable. When the induction of TI is successful, the interval between the removal of restraint and spontaneous righting is commonly used as a dependent variable. However, since some animals may sustain TI for prolonged durations, many researchers apply a 600-s cut-off, after which they gently prod the animal to conclude the episode.

The presence of either a human experimenter or a taxidermied natural predator has been used in several experiments to simulate environmental threats. For example, Gallup et al. (1971a) manually immobilized chicks at four different distances from a stuffed Cooper’s hawk. They observed a significant negative correlation between the distance from the hawk and the time spent in TI; that is, the closer the chicks were to the hawk, the longer the time they spent in TI. In a second experiment, chicks in a control group were immobilized without the stuffed hawk, while chicks in another group were held in front of the hawk before being immobilized. An additional group of chicks received the same treatment as the second group, except that a black hood covered the hawk’s face. Chicks immobilized in front of the unhooded hawk required significantly fewer attempts for induction before entering TI and remained immobile significantly longer than those exposed to the hooded hawk, which required significantly more induction attempts and stayed in TI significantly shorter than the controls.

The results of the second experiment suggest that the looming figure of the hawk enhances TI; however, the most significant stimuli are associated with its head. This led to the hypothesis that covering only the eyes of the taxidermized hawk would decrease susceptibility to and duration of TI. This hypothesis was supported. Chicks immobilized in front of the hawk with their eyes covered by tape were considerably less susceptible to TI and experienced notably shorter TI durations than chicks immobilized in front of the hawk with uncovered eyes. Moreover, Gallup et al. (1971b) found that chicks immobilized under a pair of glass eyes were significantly more susceptible to TI and demonstrated significantly longer durations of TI compared to chicks in control groups. Arduino and Gould (1984) replicated the effect of exposure to glass eyes on TI in chicks, a phenomenon that has also been observed in green anoles (Hennig, 1977) and blue crabs (O'Brien and Dunlap, 1975).

Burghardt and Greene (1988) conducted a replication study examining the effects of a simulated predator’s presence and exposure to artificial eyes on 13-day-old Eastern hognose snakes. However, their methodology for inducing responses differed; in addition to the use of TI, their observations included several strike responses exhibited by the snakes in reaction to a human approach. Two distinct experiments were performed. In both cases, the investigator initially approached the snake and leaned over to a distance of 25 centimeters. After 10 s, the investigator made contact with the snake, followed by light stroking 10 s thereafter. If none of these actions prompted the snake to exhibit death-feigning behavior, the specimen was gently picked up and shaken without being inverted. All six snakes demonstrated inversion and assumed an immobile state at some point during the induction process.

In Experiment 1, the snakes were tested both in the presence of a stuffed adult screech owl and without the owl. Experiment 2 consisted of three conditions for the snakes. In Condition 1, the experimenter stared directly at the snake from a distance of 1 m. In Condition 2, the experimenter remained in the same spot, but his eyes were turned away. In Condition 3, the experimenter moved out of the snake’s line of sight.

The snakes’ behaviors were observed during the bluff phase, the death feign, and recovery from TI. Full recovery from TI was noted when the snake crawled away from the location where it had become immobile. Several behaviors were observed between the end of the complete motionlessness characteristic of TI and the start of crawling. These included “retracting the tongue, closing the mouth, the first tongue flick, raising the head, turning the head over, bouts of tongue flicking, and partial to full righting of the body” (Burghardt and Greene, 1988, p. 1842). The latency between the onset of TI and the appearance of each of these behaviors during recovery was recorded.

The latency between entering TI and initiating crawling was significantly longer in the presence of a stuffed adult screech owl compared to its absence. In Experiment 2, the time taken from entering TI to initiating crawling was significantly extended when the experimenter was observing the snake, as opposed to when his eyes averted. When the experimenter was not present, this latency was noticeably shorter than in the other two conditions. Additionally, in Experiment 2, the snakes exhibited significantly more behavioral patterns during recovery when the experimenter was present, regardless of whether he was gazing at the snake or had his eyes averted. It should be noted that the only significant effects found in this study relate to TI and its termination. Therefore, this study does not support claims that, aside from TI, the behaviors associated with death feigning provide any protection from predation.

Manipulating the imminence of specific threatening stimuli influences TI. Similarly, manipulating the imminence of refuge-associated stimuli has an effect. For instance, Hennig et al. (1976) induced TI in green anoles situated either on an empty table or on one adorned with a natural habitat for the lizards, specifically aspidistra leaves placed near the immobilization site. The experimenter remained in the lizard’s view throughout the testing session. Anoles immobilized in the presence of aspidistra foliage demonstrated significantly shorter TI durations compared to those immobilized on the empty table.

Ewell et al. (1981) extended these findings to rabbits by observing a distance-related effect of both the experimenter and the home cage on TI. In one experiment, the investigator either moved out of sight or sat at one of three distances from a rabbit where TI had been induced. In a second experiment, TI was induced in the presence of the rabbit’s home cage, which was placed 40, 120, or 240 cm away. The investigator moved out of sight after inducing TI. In the fourth condition, neither the investigator nor the home cage was visible after TI was induced. A significant effect of distance between the experimenter and the rabbit was observed. The further the experimenter was from the rabbit, the shorter the duration of TI. There was also a significant effect regarding the distance of the home cage from the rabbit. When the home cage was 40 cm away, the rabbits recovered significantly more quickly from TI than when the home cage was at either of the other distances or absent altogether. Additionally, after righting itself, the rabbit frequently hopped into its cage. Under all other conditions in these experiments, the rabbits did not leave the trough after righting, suggesting that when the home cage refuge was nearby, the risk of flight was assessed as minimal.

Sargeant and Eberhardt (1975, p. 111) observed that, while in TI, ducks lift their heads and look around, suggesting they are “alert and aware of escape opportunities.” Visual and possibly auditory cues from nearby foxes appeared to delay recovery. Laboratory experiments support this finding. The presence of a surrogate predator (either a human or a stuffed bird of prey) significantly increases TI duration across several species (Arduino and Gould, 1984; Burghardt and Greene, 1988; Ewell et al., 1981; Gallup et al., 1971a; Hennig, 1977; O'Brien and Dunlap, 1975), and there is an inverse relationship between the duration of TI and the distance from the immobile prey to the surrogate predator (Ewell et al., 1981; Gallup et al., 1971a). Furthermore, the presence of objects associated with refuge near immobile prey significantly reduces TI duration (Ewell et al., 1981; Hennig et al., 1976). One interpretation of these results is that a prey animal in TI continuously monitors the predator’s actions, including its proximity and orientation toward the prey, as well as the availability of refuge. If the immobile prey observes that the predator is nearby and looking at it and that refuge is either distant or unavailable, threat imminence is high, along with the risk of transitioning from the circa-strike TI response to post-encounter flight. Consequently, the TI episode is prolonged.

This interpretation is framed by the risk assessment and predator imminence hypotheses of prey behavior. Therefore, it is not entirely theory-neutral. However, it offers an explanation that refers only to observed behavior. There is no mention of mental states such as beliefs or desires. According to Dennett’s (1983) classification, it is an explanation based on a zero-order intentional system.

Burghardt (1991) expands this interpretation by arguing that these experiments, particularly those conducted by Burghardt and Greene (1988), provide evidence for the existence of higher-level intentional systems in prey animals. His argument may have merit. As Dennett suggests, taking “a risky step into intentional characterization” could yield “gains in perspicuity, in predictive power, in generalization” (Dennett, 1983, p. 347). A modest step would be to hypothesize that a first-order intentional system is associated with TI. Under this hypothesis, the prey either remains in or terminates TI because it *believes,* based on its assessment of the predator’s position and posture, that the predator poses a varying level of threat to it and that certain objects in the environment, when present, relate *to its* safety.

The association of a first-order intentional system with target-induced (TI) responses incorporates cognitive processes into what might otherwise be a purely behavioral interpretation of these reactions. This enhancement may appear unnecessary to clarify the onset of TI. In the context of evasive maneuvers to avoid predation, circa-strike behaviors must represent immediate reactions to potentially catastrophic encounters. These behaviors are primarily influenced by direct sensory stimuli, circumventing elaborate forebrain processing (Fanselow, 1994, p. 435). Given that TI activates when a predator successfully captures its prey, its initiation aligns precisely with Fanselow’s characterization of a circa-strike response. Therefore, there appears to be little merit in suggesting a more complex system than the presence of a zero-order intentional system within the prey to explain the onset of TI. Nonetheless, findings from the previously mentioned experiments indicate that the cessation of TI involves a more intricate sensory processing mechanism than its initiation. The existence of a first-order intentional system would provide the prey with a cognitive framework for interpreting various environmental conditions and formulating adaptable behavioral responses (Ristau, 1991). Thus, the assertion that the termination of TI is associated with activities within a first-order intentional system may lead to more accurate predictions regarding the impacts of changing environmental stimuli than a simplistic zero-order behavioral hypothesis.

However, Burghardt takes an additional step. He hypothesizes that the research mentioned above supports, at the very least, the existence of a second-level intentional system. A second-level intentional system suggests that the prey possesses beliefs about its own intentional mental states as well as those of its predator. For instance, during the bluff stage of the circa-strike mode, the hognose snake *aims for* the predator to *believe* it is a venomous viper and, during the threat indication, that it is dead. Nevertheless, it *fears* that the predator may not be fooled (Burghardt, 1991, Table 4.6, p. 76). This hypothesis leads to the conclusion that the use of subtle visual cues by a prey animal to adaptively modify its behavior “is an example of mind reading” (Burghardt, 1991, p. 78).

It is unclear how this hypothesis enhances clarity, predictive power, or generality compared to hypotheses that only involve lower-level intentional systems. The hypothesis that TI is a species-typical predator defense response requiring minimal cognitive processing (a zero-order intentional system) appears adequate to explain its initiation. The idea that the prey animal recognizes or perceives the predator as a threat, understanding that increased distance or the presence of structures indicating safety reduces that threat (a first-order intentional system), sufficiently explains the termination of TI. Suggesting that altering the duration of TI through manipulations of predator surrogates constitutes evidence of a second-level intentional system appears to be an unnecessary violation of Morgan’s canon.

Acceptance or rejection of the second-level intentional system hypothesis has implications for Burghardt’s (1991) assertion that animals’ use of visual cues in the theory of mind (TI) represents an instance of mind reading. Furthermore, since mind reading is associated with consciousness, this is relevant to the relationship between TI and consciousness. However, these implications depend on Burghardt’s definition of mind reading, which he does not provide. If he refers to minimal mind reading as described by Bermúdez (2009), it could relate to a first-order or even zero-order intentional system. According to Bermúdez (2009, p. 146): “A creature engages in *minimal mind reading* when its behavior is systematically dependent upon changes in the psychological states of other participants in the interaction.” There is no requirement for the animal participating in minimal mind reading to represent the mental (psychological) state of the other participant.

However, this does not appear to be the type of mindreading Burghardt intended. Instead, his examples indicate that prey in TI engages in substantive mindreading. Citing Bermúdez (2009, p. 146): “Attributions of substantive mindreading are made to explain how and why an animal’s behavior systematically depends on the psychological states of other participants in the interaction. Those who identify substantive mindreading in the animal kingdom typically argue that what explains this dependence is the fact that the animal involved in a social interaction mentally represents the psychological states of the other participants.”

As previously argued, the outcomes of research into TI can be interpreted without suggesting that either the predator or the prey has a mental representation of the psychological state of the other participant. Therefore, I reject the hypothesis that TI implies substantive mindreading. Since substantive mindreading involves at least a second level, and potentially a third level, of intentional states, I also reject the hypothesis that TI signifies the existence of these higher-order intentional states, along with the related hypothesis that they are useful in explaining TI (Burghardt, 1991).

## 6 Implications for phenomenal consciousness

### 6.1 Anoetic, noetic, and autonoetic consciousness

In the preceding section, I concluded that the initiation of TI is a reflex response to predator restraint, requiring no more than a zero-order intentional representation of danger. However, the results of the experiments reviewed above can reasonably be interpreted as demonstrating that mental representation of safe escape routes and viable shelter significantly influences when a TI episode is terminated. Therefore, invoking a first-order intentional representation provides a more complete explanation for the termination of TI than relying solely on a zero-order intentional representation.

If the presence of intentional content is a precondition for phenomenal consciousness (Gennaro, 2018), and if the intentional mental representation of the qualities of interoceptive and exteroceptive stimuli plays a role in TI, it may be that TI indicates the existence of phenomenal consciousness. However, the specific type of phenomenal consciousness that is present remains an unresolved question. Drawing from a theory first proposed by Tulving (1985), Vandekerckhove and Panksepp (2009) provide a valuable context in which this question may be explored. They suggest that consciousness exists along a three-stage evolutionary continuum consisting of anoetic consciousness, noetic consciousness, and autonoetic consciousness.

Anoetic consciousness corresponds to primal perceptual consciousness, as described by Merker (2007) and Feinberg and Mallatt (2016). It is evolutionarily ancient, entirely experiential, and unreflective. Consequently, it may provide a useful conceptual framework for interpreting the sequence of behaviors involved in the predator imminence cascade. These behaviors are species-typical, and if influenced by experience, they are modified by implicit procedural, sensory, and affective memory processes (Panksepp, 2005; Vandekerckhove and Panksepp, 2009). Thus, anoetic consciousness provides an adequate conceptual framework, at least for the initiation of TI.

However, in certain contexts, the cessation of a TI episode in prey animals is influenced by their assessment of environmental threats or safety levels. This observation suggests the potential involvement of a more intricate level of consciousness. Such a level of consciousness requires the ability to utilize knowledge acquired from general facts stored in memory concerning the present circumstances. In non-human animals, the retention of general facts has been associated with semantic memory (Martín-Ordás, 2021), a type of memory associated with noetic consciousness (Vandekerckhove and Panksepp, 2009; Tulving, 1985). Consequently, noetic consciousness may provide a broader framework for elucidating the termination of TI episodes. Nevertheless, this contextual understanding does not require episodic memory, reflective self-awareness, or autonoetic consciousness.

### 6.2 The fear hypothesis of TI

As indicated in the introduction, an alternative hypothesis is associated with TI, which may have significant implications for phenomenal consciousness. Proponents of this hypothesis argue that fear is inextricably linked to TI (Carli and Farabollini, 2022b; Gallup, 1977; Jones, 1996). If empirical evidence is obtained to support the fear hypothesis of TI, and if it is accepted that fear involves a subjective state, one might argue that animals exhibiting TI possess the capacity for at least anoetic consciousness.

Gallup (1977), Jones (1987), and Carli and Farabollini (2022b) have suggested that, while not the cause of TI, fear influences it significantly. The studies reviewed in the previous section can be interpreted as supporting this “fear hypothesis” of TI. For example, when the predator is close to the prey animal and observing it, the prey is presumably more fearful than when the predator is at a distance and its eyes are not visible. Thus, the closer the predator, the longer the prey remains in TI. Conversely, the presence of a nearby refuge likely reduces fear and shortens the duration of TI.

### 6.3 Experimental evidence for the fear hypothesis of TI

Support for the fear hypothesis also stems from various experiments specifically designed to test it. For instance, gentle handling repeated in the weeks leading up to testing for TI, which presumably reduces fear of humans, correlates with decreased susceptibility to and duration of experimenter-induced TI in guinea pigs (de Lima Rocha et al., 2017) and chickens (Gallup, 1979; Gilman et al., 1950). In contrast, rough handling immediately before induction increases TI in chickens (Jones, 1992). Furthermore, when other inherently aversive stimuli thought to induce fear are presented before TI induction, they increase the susceptibility and duration of TI. These stimuli include electric shock (Edson and Gallup, 1972; Gallup et al., 1970a, 1970b), loud noise (Gallup et al., 1970b; Nash et al., 1970), or suspension over a visual cliff (Gallup and Williamson, 1972). Presenting a conditioned stimulus that indicates impending shock also enhances TI (Gallup et al., 1972; Gallup, 1973; Maser et al., 1973). Conversely, presenting a CS that indicates the absence of shock reduces its duration (Maser et al., 1973). Finally, TI has a positive correlation with other presumed measures of fear, such as freezing in an open field or novel environment, emerging into an open area from a closed home base, and home cage avoidance tests {Jones, 1987, 1996; Jones and Waddington, 1992}.

The relationship between TI and fear has been widely acknowledged (e.g., Carli and Farabollini, 2022d; de Lima Rocha et al., 2017; Leite-Panissi, 2001; Moskowitz, 2004; Prestrude, 1977). Indeed, TI is even used as an indicator of fear in the poultry industry (Forkman et al., 2007; Jones, 1987; Jones et al., 1981; Minvielle et al., 2002; Sumanu et al., 2019; Zulkifli et al., 2009; Carli and Farabollini, 2022e). However, a challenge remains regarding the fear hypothesis: the ambiguity surrounding what the term “fear” is meant to convey.

### 6.4 Fear as an intervening variable

This lack of clarity leads to disagreements among prominent scientists studying fear (Mobbs et al., 2019). For example, Joseph LeDoux contends that **“**subjective states of fear should not be invoked to describe the defensive behavior of species in which such states cannot be verified by verbal report” (LeDoux et al., 2017, p. 27). Thus, defensive behaviors such as freezing, fleeing, and fighting with animals should not be equated with fear (LeDoux, 2013, 2014). Instead, they should be referred to as ‘threat-elicited defense responses.’ Embracing LeDoux’s perspective suggests that the fear hypothesis of TI is incoherent and should be dismissed.

On the contrary, Michael Fanselow rejects the definition of fear that is limited to subjective experience. For Fanselow, fear refers to “*the activation of the defensive behavioral system* that gives rise to this constellation of reactions to threatening stimuli” (Fendt and Fanselow, 1999, p. 743, italics added). This constellation of reactions includes autonomic, somatomotor, and subjective cognitive-emotional components (Fanselow and Pennington, 2018). Fanselow’s conceptualization of fear is compatible with the fear hypothesis of TI proposed by Gallup (1977), Gallup (1979), which defines “fear as an intervening variable, not a hypothetical construct” (Gallup, 1977, p. 58).

MacCorquodale and Meehl (1948) provide an insightful conceptualization of an intervening variable that may aid in understanding the relationship between fear—used by Gallup and in this paper—and TI, along with other threat-related behaviors. An electron, while not directly observable, is based on inferential evidence and is considered a postulated entity, which MacCorquodale and Meehl highlight as an example of a hypothetical construct. Resistance, on the other hand, is a measurable relationship between current (amperage) and electromotive force (voltage), yet it remains neither directly observable nor an entity. Instead, it represents a fundamental property of a specific material.

Using this analogy, I can see that fear is viewed as a feature of the neural systems that govern specific responses to particular stimuli, and its intensity can be gauged through nonverbal or verbal behavior. For example, in the study by Ewell et al. (1981), the intensity of fear correlates with the time spent in the test area (TI) in the presence of either the experimenter (E) or the home cage (HC), divided by one of the three distances (D) between the rabbit and E or HC (F = P/D or F = HC/D). This example does not intend to imply that fear can be accurately measured in the same systematic way that Ohm’s law measures resistance; instead, it demonstrates how fear might be understood as an intervening variable.

In conclusion, as intended by Gallup, the classification of the term “fear” as an independent variable facilitates its application as a heuristic framework for formulating hypotheses regarding the mechanisms that underpin TI and for designing experiments aimed at testing these hypotheses, all while obviating the necessity for specific definitions pertaining to subjective fear in non-human species. Nevertheless, the possibility of including such definitions remains open. Consequently, this methodology for defining fear allows, when beneficial for the development of hypotheses, the inclusion of references pertaining to anoetic, noetic, and autonoetic consciousness.

## 7 Discussion

In this study, I review several claims about TI. One claim suggests that animals exhibiting TI are merely pretending to be genuinely dead. This claim implies that predators prefer to attack and consume live prey, abandoning the prey animal if they perceive it as dead. To contest this assertion, I first differentiate between TI and death feigning. Unlike TI, death feigning has a relatively limited range within the animal kingdom, even among members of the same class. For instance, in a comprehensive review of the behavior of 52 snake species from North, Central, and South America, Magallón et al. (2021) reported that 33 species demonstrated TI, while only 13 exhibited three or more of the other behaviors typically associated with death feigning. In fact, substantial variation in the relative prevalence of TI and death feigning can be found within the same family. For example, the Eastern hognose snake (*Heterodon platirhinos*) and the Patagonia green racer (*Philodryas patagoniensis*) are both rear-fanged, mildly venomous snakes from the family Colubridae. However, the Eastern hognose displays multiple death-feigning behaviors, while the Patagonia green racer exclusively shows TI (Magallón et al., 2021). This variation in species distribution between death-feigning and TI indicates differences in evolutionary mechanisms.

### 7.1 Death feigning as a parasympathetic response

Furthermore, while evidence shows that TI increases the likelihood of prey escaping predation, the various responses associated with TI that occur during death-feigning have not been shown to provide such protection. However, these responses typically follow active kinetic behaviors associated with post-encounter predator avoidance (e.g., flight) and the initial circa-strike phase (e.g., baring teeth and growling in the opossum, bluffing in the Eastern hognose snake). Conversely, TI is a passive, akinetic behavior. As McDonald (1974, p.161) noted regarding the death feign of the hognose snake: “Except for writhing and inversion of the body, the common denominator of these events is not that they generally occur in dying snakes and would contribute to an accurate simulation, but rather that they, like bradycardia, are parasympathetic effects.” This observation also applies to the opossum (Francq, 1969; Gabrielsen and Smith, 1985). Thus, most instances of death feigning, rather than serving as a predator defense function, may represent a shift from an active to a passive form of predator defense.

Therefore, borrowing from Hartman (1950, p. 53), death feigning and its associated term thanatosis are mere “shortcuts to mental satisfaction” and represent a “characteristic of the unscientific and uncritical treatment of any subject.” Consequently, I propose that, just as the term “animal hypnosis” has largely been phased out, death feigning and thanatosis should also be abandoned, possibly replaced with the term parasympathetic response pattern (PRP).

### 7.2 TI and intentionality

A second claim primarily articulated by Burghardt (1991) and also supported by Ristau (1991) is that death feigning demonstrates that animals can have intentional states. Burghardt contends that the entire death-feigning behavior is essential to uphold his hypothesis; however, the only evidence he presents to back it is changes in the duration of TI. Consequently, death feigning appears to be irrelevant to evaluating his hypothesis. Nonetheless, these data may be utilized to support the intentional stance hypothesis in a limited capacity.

This defense is based on the idea that TI consists of two components: initiation and termination. TI occurs after a predator pursues and struggles with its prey, specifically when the prey is captured and restrained. Initiation is likely, as Klemm (1969, 1971, 1977) suggested, a brainstem reflex that does not require complex sensory processing, making a zero-order intentional system a sufficient explanation. However, for TI to end in a successful escape, the prey must determine whether to remain immobile or flee based on its perception of the understanding of the predator’s behavior and access to potential refuge. Consequently, the termination of the response is more complex than initiation and involves higher cognitive processes, relying on a first-order intentional system.

### 7.3 TI is driven by subcortical neural circuits, especially those found in the brainstem

The neocortex does not appear to be essential for TI. As described above, chickens (Arduino and Gould, 1984; Gallup et al., 1971a, 1971b), green anoles (Hennig, 1977; Hennig et al., 1976), and Eastern hognose snakes (Burghardt and Greene, 1988), which lack a six-layered neocortex, have been shown to assess their environment for the presence of predator surrogates or shelter while in TI. Furthermore, in rabbits, a species with a developed neocortex that is susceptible to TI, neither lesions limited to the sensory-motor cortex (Woodruff et al., 1975) nor complete decortication (Carli, 1969a; Carli and Farabollini, 2022f) impact TI.

Furthermore, a unique cortical EEG signature is not associated with TI. Low-voltage fast-wave EEG activity, characteristic of attentive wakefulness, occurs prior to the onset of TI and continues afterward in rabbits (Carli, 1969b; Carli and Farabollini, 2022f; Harper, 1971; Hatton et al., 1975; Klemm, 1969, 1971; Svorad, 1957; Whishaw et al., 1982) and opossums (Barratt, 1965; Norton et al., 1964). In rabbits, when TI lasts longer than 4–5 min, large irregular slow waves, typical of later sleep stages, have been observed in the cortical EEG. Low-voltage fast waves reappear just before TI termination (Carli and Farabollini, 2022f; Harper, 1971; Hatton et al., 1975; Klemm, 1971; Whishaw et al., 1982).

It should be noted that the lack of effect from neocortical lesions and the absence of a unique EEG pattern in species susceptible to TI do not necessarily indicate that the neocortex is uninvolved in the response. For instance, Klemm (1971, 1977) hypothesized that the neocortex inhibits TI, which explains its absence in many mammals. There is some, albeit limited, support for this hypothesis. For example, laboratory rats show significant resistance to TI, demonstrating it only in a few trials, with durations averaging less than 10 s when it does occur (McGraw and Klemm, 1969; Prestrude, 1977; Teschke et al., 1975; Webster et al., 1981; Woodruff and Bailey, 1979). The application of KCl increases the mean duration of TI in rats, but only to an average of 19.4 s compared to 1.43 s in untreated controls (Teschke et al., 1975). Furthermore, McGraw and Klemm (1969) showed that decortication significantly extends TI duration in rats, but this effect lasts only 5 or 6 postoperative days.

Therefore, even these effects of neocortical manipulation do not support a significant role for the neocortex in TI. However, classic limbic system structures—particularly the amygdala (de Paula et al., 2019; de Paula and Leite-Panissi, 2016; Donatti and Leite-Panissi, 2009; Leite-Panissi et al., 2003; Leite-Panissi et al., 2006; Leite-Panissi and Menescal-de-Oliveira, 2002; Leite-Panissi et al., 1999)—as well as the septal area (Woodruff and Lippincott, 1976), hippocampus (Woodruff et al., 1975), and anterior cingulate cortex (Woodruff et al., 1981) modulate TI, and in intact animals, these structures presumably play an important role in its effectiveness as predator defense. The medulla, pons, and midbrain, however, provide the necessary and sufficient neural circuitry for its initiation and termination. This claim is supported by the observation that TI, physically identical to that in intact rabbits, can be elicited in rabbits with transverse pre-collicular brainstem sections (Carli, 1969a; Carli, 1977). Assuming that telencephalic brain structures are required for noetic and autonoetic phenomenal consciousness in animals that normally possess them, Carli’s experiments demonstrate that the occurrence of TI does not serve as evidence for the presence of these higher-order processes, nor are they necessary to explain TI. However, a network of structures in the brainstem is essential for TI, likely providing an adequate substrate for anoetic/primal sensory phenomenal consciousness (Feinberg and Mallatt, 2016; Merker, 2007; Vandekerckhove and Panksepp, 2009; Woodruff, 2017).

The pontomedullary reticular formation (PMRF) is a crucial component of this network. It receives ascending input from the anterior lateral spinal pathways, which convey poorly localized “crude” touch and nociception (Fields et al., 1977; Menetrey et al., 1980), likely serving as the primary sensory input that initiates TI. Furthermore, the PMRF gives rise to reticulospinal tracts that exert bilateral influence over spinal cord motor circuits controlling axial and proximal limb muscles, often engaging both upper and lower limbs simultaneously (Jones and Yang, 1985; Yezierski, 1991). Thus, the PMRF is well-positioned to play a significant role in controlling TI. This hypothesis is supported by Klemm’s observations of a correlation between multi-unit activity in the PMRF and TI (Klemm, 1969, 1971) and by findings that electrical stimulation of the PMRF significantly increases the duration of TI (Klemm, 1965) in rabbits.

The midbrain periaqueductal gray (PAG) is a crucial brainstem structure involved in both the initiation and termination of TI. Experiments conducted on guinea pigs by neuroscientists at the University of São Paulo implicate the dorsal periaqueductal gray (dPAG) in the termination of TI (Monassi et al., 1997) and the ventrolateral periaqueductal gray (vlPAG) in its initiation and maintenance (Coutinho et al., 2008; Monassi et al., 1997; Monassi et al., 1999; Vieira-Rasteli et al., 2018). These findings align with observations that the dPAG is associated with other kinetic predator defense behaviors, such as flight, jump attacks, and fighting (Bandler and Shipley, 1994; De Oliveira et al., 2001; Leiras et al., 2022; Fanselow, 1994; Fanselow et al., 1995) while the vlPAG is associated with freezing in response to threatening stimuli (Bandler and Shipley, 1994; de Mello Rosa et al., 2022; Fanselow et al., 1995; Ferreira-Pinto et al., 2018; Reis et al., 2023; Tovote et al., 2016).

Several other brainstem structures have been associated with TI in guinea pigs, most notably the parabrachial nucleus (Menescal-de-Oliveira and Hoffmann, 1993), the nucleus raphe magnus (da Silva and Menescal-de-Oliveira, 2007; Ferreira and Menescal-de-Oliveira, 2012), and the dorsal raphe nucleus (Ferreira and Menescal-de-Oliveira, 2009, 2012). Discussing the research that details the interactions among these nuclei and their associated neurotransmitters is beyond the scope of this review, as is exploring the relationship of these structures to TI and pain. Such discussions are also unnecessary, as excellent reviews outlining the neural basis of TI have recently been published by Carli and Farabollini (2022g) and Lalonde and Strazielle (2022), and the topic of TI and pain has been well presented by Carli and Farabollini (2022h).

In summary, the neurological evidence reviewed in the previous section supports the claim that TI is linked to anoetic consciousness. However, Carli’s (1969b, 1977) research on the effects of decerebration in rabbits regarding TI has been interpreted to suggest a lack of involvement of telencephalic structures in the response. This could negate any role for noetic consciousness in TI. However, as previously mentioned, the amygdala, anterior cingulate cortex, hippocampus, and septal nuclei modulate TI. In intact rabbits, these structures may help monitor environmental stimuli that signal either threat or safety. This could provide a level of response flexibility not available to decerebrate rabbits and allow for a more advanced noetic level of consciousness.

This suggests that TI is the result of evolution. Indeed, TI is generally considered to be an evolved predator defense and has also been proposed as an evolutionary antecedent of certain human higher-order mental states. The next section considers the evidence for these proposals.

### 7.4 TI, evolution, and theory of mind

TI demonstrates two essential prerequisites for evolution: variability in response within a species and heritability. For example, Gallup (1974b) found that both long and short TI durations underwent significant artificial selection in chickens. Jones et al. (1991) observed a similar effect in Japanese quail, while McGraw and Klemm (1973) noted modest artificial selection for TI duration in rats. As outlined in recent reviews by Carli and Farabollini (2022c, 2022i), these two prerequisites for evolution have proven beneficial in demonstrating fitness differences related to TI. For example, Miyatake et al. (2004) showed that red flour beetles selected for longer TI durations were more successful in evading predation by Adanson jumper spiders compared to those selected for shorter durations.

However, I argue that caution is warranted when extrapolating evidence for the adaptive evolution of timing intervals (TI) to support theories proposing that TI serves as the evolutionary basis for certain cognitive functions in humans. An example of such extrapolation is provided by Tsoukalas (2018), who suggested that TI is the evolutionary foundation of human theory of mind (ToM). He presents several claims to strengthen his hypothesis. One claim of central importance is that TI is “predicated on cholinergic neurotransmission” (Tsoukalas, 2018, p. 50), yet he only cites a paper by Thompson (1977) to substantiate this assertion. In the referenced paper, Thompson detailed original research conducted by Thompson et al. (1974), demonstrating that the competitive cholinergic inhibitor scopolamine significantly diminished TI in chickens. This effect has been replicated in chickens (Hennig et al., 1988; Gagliardi and Thompson, 1977; Sanberg, 1983) and extended to ducks (Woodruff et al., 1976). Conversely, the acetylcholinesterase inhibitor physostigmine was shown to increase the duration of TI in both chickens (Thompson et al., 1974) and ducks (Woodruff et al., 1976), while pilocarpine, a cholinergic receptor agonist, also extended TI duration in chickens (Sanberg, 1983). The findings from these experiments strongly support the hypothesis that cholinergic transmission enhances TI in avian species.

However, Tsoukalas (2018) failed to cite experiments that raise substantial concerns about the generalizability of his hypothesis to mammals. In contrast to the effects noted for chickens and ducks, Hatton et al. (1975) found that scopolamine significantly increased the duration of time intervals (TI) in rabbits, while physostigmine markedly reduced TI duration. Woodruff et al. (1976) replicated these results in guinea pigs. Therefore, the hypothesis that TI is “predicated on cholinergic neurotransmission” is only supported by birds. To the extent that the evolutionary origin of a specific phenotype in humans can be linked to a phenotype observed in animals, it appears reasonable to suggest that the closer the genetic relationship between humans and animals, the more likely this connection exists. Thus, based on the studies just discussed, if there is any relationship between activity in central cholinergic pathways, TI, and human Theory of Mind (ToM), it appears to falsify rather than support Tsoukalas’s hypothesis.

## 8 Conclusion

In this study, I reviewed the claim that animals exhibiting TI are doing so to intentionally feign death, as well as the related assertion that this deceptive behavior indicates the presence of third- and fourth-level intentional systems. I also examined the so-called fear hypothesis of TI. In each instance, while not adopting a strictly behavioristic perspective, I countered an interpretation of the data that relied on what Burghardt (1991) referred to as critical anthropomorphism. Research on the factors influencing TI and its neural mechanisms should steer clear of the bias that Branch and Malagodi (1980) discussed, which often accompanies the terminology of animal hypnosis, death feigning, and thanatosis.
